# γ-Tocotrienol as a Promising Countermeasure for Acute Radiation Syndrome: Current Status

**DOI:** 10.3390/ijms17050663

**Published:** 2016-05-03

**Authors:** Vijay K. Singh, Martin Hauer-Jensen

**Affiliations:** 1 Department of Pharmacology and Molecular Therapeutics, F. Edward Hébert School of Medicine, Armed Forces Radiobiology Research Institute, Uniformed Services University of the Health Sciences, Bethesda, MD 20889, USA; 2 Department of Pharmaceutical Sciences, University of Arkansas for Medical Sciences and Central Arkansas Veterans Healthcare Systems, Little Rock, AR 72205, USA; mhjensen@uams.edu

**Keywords:** γ-tocotrienol, granulocyte colony-stimulating factor, mice, neutropenia, nonhuman primates, radiation, thrombocytopenia

## Abstract

The hazard of ionizing radiation exposure due to nuclear accidents or terrorist attacks is ever increasing. Despite decades of research, still, there is a shortage of non-toxic, safe and effective medical countermeasures for radiological and nuclear emergency. To date, the U.S. Food and Drug Administration (U.S. FDA) has approved only two growth factors, Neupogen (granulocyte colony-stimulating factor (G-CSF), filgrastim) and Neulasta (PEGylated G-CSF, pegfilgrastim) for the treatment of hematopoietic acute radiation syndrome (H-ARS) following the Animal Efficacy Rule. Promising radioprotective efficacy results of γ-tocotrienol (GT3; a member of the vitamin E family) in the mouse model encouraged its further evaluation in the nonhuman primate (NHP) model. These studies demonstrated that GT3 significantly aided the recovery of radiation-induced neutropenia and thrombocytopenia compared to the vehicle controls; these results particularly significant after exposure to 5.8 or 6.5 Gray (Gy) whole body γ-irradiation. The stimulatory effect of GT3 on neutrophils and thrombocytes (platelets) was directly and positively correlated with dose; a 75 mg/kg dose was more effective compared to 37.5 mg/kg. GT3 was also effective against 6.5 Gy whole body γ-irradiation for improving neutrophils and thrombocytes. Moreover, a single administration of GT3 without any supportive care was equivalent, in terms of improving hematopoietic recovery, to multiple doses of Neupogen and two doses of Neulasta with full supportive care (including blood products) in the NHP model. GT3 may serve as an ultimate radioprotector for use in humans, particularly for military personnel and first responders. In brief, GT3 is a promising radiation countermeasure that ought to be further developed for U.S. FDA approval for the ARS indication.

## 1. Introduction

Nuclear and radiological mass-casualty incidents are significant risks to deployed military members and civilian populations. Disasters, such as the Fukushima Daiichi Nuclear Power Plant and the Chernobyl Nuclear Power Plant, emphasize the need for available and effective therapeutic options to mitigate and treat the damaging effects of exposure to lethal doses of ionizing radiation and combined injury [1,2,3]. There are currently more than 100 countries without adequate monitoring systems or regulatory control in place to discourage terrorist groups from obtaining radioactive material, detonating a dirty bomb or using radiological dispersal devices; therefore, the nation must plan for the inevitability of such an event in the future [4]. In the worst possible situation, an improvised nuclear device, radiological dispersal device or dirty bomb would be detonated in a densely-populated city, inciting not only panic, fear, injury and death, but also an array of other medical issues arising from the blast, radioactive fallout and extreme heat. The large number of individuals affected by these events would require urgent medical attention and management [5,6].

In humans, substantial acute radiation injury occurs at radiation doses above 1 Gray (Gy), with indications getting increasingly severe with the increase of absorbed radiation dose [7]. After an exposure in the range of 1 to 6 Gy, the hematopoietic system is damaged in a dose-dependent manner, resulting in hematopoietic acute radiation syndrome (H-ARS), which is associated with severe damages to the hematopoietic system. The numbers of white and red blood cells, platelets, neutrophils, lymphocytes and other blood cell components drop and the vulnerability to possibly lethal infections significantly increases. After exposure to ~6 to 8 Gy, hematopoietic symptoms are still present, though with them arise additional symptoms caused by the breakdown of the gut mucosa, resulting in gastrointestinal syndrome (GI-ARS). GI syndrome includes severe injury to the GI tract and translocation of gut bacteria to peripheral circulation and to remote organs, which results in sepsis and ultimately death of the radiation-exposed victim. H-ARS and GI-ARS are well accepted as the major sub-syndromes of ARS. After exposure to even higher radiation doses, significant, irreversible damage occurs in the nervous and cardiovascular systems (known as neurovascular syndrome), resulting in unconditional and rapid death [7]. Because of the degree of damage and the rapidity of symptom onset, neurovascular syndrome has been regarded as untreatable by the clinical and scientific research community, and therefore, efforts have been focused on discovering preemptive and mitigating therapy for H-ARS and GI-ARS victims. The search for suitable radiation countermeasures has been ongoing for several decades and has resulted in the identification of various categories of radiation countermeasures, which can be used as radioprotectors or mitigators [8,9,10,11]. Several novel approaches, including cell-based therapies, have been investigated [9,12,13,14,15,16,17]. However, to date, only two radiomitigators for H-ARS and no radioprotector have been approved by the U.S. Food and Drug Administration (U.S. FDA) for the treatment or prevention of ARS [18,19,20]. Amifostine (WR2721) is another radioprotector that has received U.S. FDA approval for narrowly-defined indications in humans [21,22,23,24]. Amifostine is clinically used for the reduction of xerostomia (dry mouth) resulting from salivary gland injury in head and neck cancer patients undergoing radiotherapy and to protect against renal toxicity as a result of cis-platinum chemotherapy to patients with advance ovarian cancer, among several other treatment modalities [25].

On many medical research fronts, natural products have been studied for the prevention and treatment of several human ailments. Such agents are generally recognized as safe (GRAS) and suitable for medicinal use; they are well accepted and have negligible toxicity, even when used in the higher ranges of food intake, compared to their synthetic counterparts [26,27,28]. Vitamin E family members are well known for their antioxidative, neuroprotective and anti-inflammatory properties and have stolen the research spotlight [28,29]. Their potent antioxidant activity helps peroxidation reactions and controls free radical generation [30,31]. As such, these agents are capable of protecting cells from enhanced oxidative damage initiated by free radicals, important mediators of radiation toxicity.

Although earlier investigations evaluated tocopherols (particularly α-tocopherol) for potential radioprotection, recent studies suggest that the therapeutic targets for tocopherols and tocotrienols are different. Such studies indicate that the vitamin E family members mostly act through diverse mechanisms and do not reveal biological attributes that notably overlap or are redundant [32,33]. Tocotrienols have clearly different functions in treating disease and maintaining health; several studies have shown superior antioxidant properties of tocotrienols compared to tocopherols [34,35,36,37,38,39]. It has been shown that δ-tocotrienol, γ-tocotrienol (GT3) and tocopherol succinate protect mice against ionizing radiation injuries [40,41,42]. Among these agents, GT3 has been most extensively investigated for its radioprotective efficacy in mice and nonhuman primates (NHPs) at the Armed Forces Radiobiology Research Institute (AFRRI) and University of Arkansas for Medical Sciences. This article provides a detailed narrative of the development of GT3 as a radiation countermeasure.

## 2. Radiation Countermeasures: Radioprotectors, Mitigators and Therapeutics

Countermeasures for radiation injury fall under three broad categories: radioprotectors, radiomitigators and radiation therapeutics. Radioprotectors are agents that are administered prior to irradiation to prevent radiation-induced molecular and cellular injuries [13,15,43]. Prophylactic agents, which include free radical scavengers, must be present at the time of irradiation, as they act during the initial radiochemical events. Radiation mitigators are agents administered after radiation exposure, but before symptoms occur, to accelerate recovery and repair of the injury caused by radiation exposure; this class includes angiotensin-converting enzyme inhibitors and growth factors. Radiation therapeutics are agents administered after overt symptoms of irradiation and appear to stimulate regeneration. Such treatments include pentoxifylline (PTX) for radiation fibrosis and cellular therapy [12,44,45,46]. Several additional candidate radiation countermeasures (specifically radioprotectors and radiomitigators) have been identified and are currently being developed following the Animal Efficacy Rule for U.S. FDA approval [8,9,28,47]. However, the state of this list is alarming; the drug candidates are few and have limited scope.

## 3. U.S. Food and Drug Administration (U.S. FDA) Animal Efficacy Rule

In 2002, the U.S. FDA issued the Animal Efficacy Rule (21 CFR Parts 314.600–650 for drugs and 21 CFR 601.90 for biological products) to expedite the development of medical countermeasures against chemical, biological, radiological and nuclear threats. This approval pathway applies only to new drugs or biologics for which human efficacy studies cannot be performed because it would be unethical to knowingly expose humans to debilitating, potentially lethal stimuli, or because field trials have not been feasible [48,49,50,51]. According to the Animal Efficacy Rule, potential products must undergo rigorous testing utilizing acceptable and well-controlled animal models to establish the safety and efficacy of the product under development. In other words, the likelihood of the product producing a clinical benefit in humans must be demonstrated before the U.S. FDA grants marketing approval. The principles of the U.S. FDA’s Animal Efficacy Rule applicable to countermeasure development using animal models are listed below:

(1) There should be a logically well-understood pathophysiological mechanism for the injury of the agent (radiation) and its prevention or significant decrease by the drug;

(2) The efficacy of the drug/biologic is established in more than one animal model likely to respond with a reaction predictive for humans, unless the efficacy is shown in one animal model that represents a satisfactorily well-characterized animal model for envisaging the response in humans;

(3) The end point of the animal study is related to an anticipated advantage in humans, generally involving the improvement of survival or the prevention of major morbidity;

(4) Pharmacokinetics, pharmacodynamics or other appropriate data or evidence of the product, in animals and humans allows for the selection of an effective human dose.

The mechanisms by which radiation causes injury still remain poorly understood (Point 1). The satisfaction of the points outlined in 2 and 3 will require good animal models with appropriate endpoints. There are many challenges faced by scientists utilizing animal models to develop radiation countermeasures; two of these involve limitations of the animal model itself. First, it is not feasible that any single animal model will be appropriate for studying all sub-syndromes of ARS or other indications. In reality, animal models seldom replicate the human disease. Therefore, the data generated from animal efficacy studies will never be as convincing as human data. Second, there are several approaches to treat radiation injuries [52], and it is highly questionable that any single animal model will be capable of adequately assessing all potential classes of agents under investigation. To ensure that an agent will be effective in humans, we must understand how and why a countermeasure may work in terms of the biological mechanism; biomarkers of both radiation injury and countermeasure efficacy are particularly important in this context [53].

When comparing to the traditional licensure pathway, the challenges and requirements of the Animal Efficacy Rule outlined above may be the reason why only a few drugs have successfully been approved utilizing this pathway. In fact, very few candidate drugs have been approved through this pathway since the FDA issued it in 2002, despite significant efforts by the federal government to encourage the development of countermeasures against potential chemical, biological, radiological and nuclear threats [54,55,56,57,58]. GT3 is currently under development following the U.S. FDA Animal Efficacy Rule.

## 4. Tocopherols and Tocotrienols: Members of the Vitamin E Family

**Figure 1:**
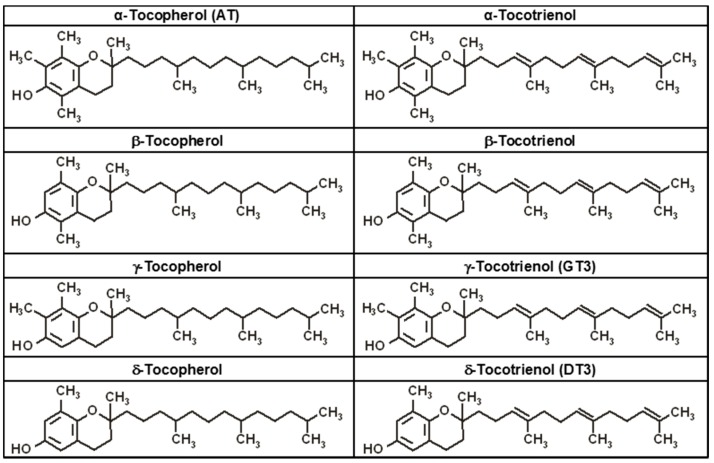
Chemical structures of all members of the vitamin E family [28].

As stated above, vitamin E represents a family of eight members known as tocols. These members act as important antioxidative agents that control free-radical generation and regulate peroxidation reactions in the body. Tocols are divided into two subgroups: tocopherols and tocotrienols. Both groups share common structural characteristics [30,31]. Tocopherols retain a 4′,8′,12′-trimethyltridecyl phytyl side chain, while the tocotrienols hold double bonds at position 3′, 7′ and 11′ in their isoprenoid tails. Each group contains four isoforms (α, β, γ and δ), characterized by the location and number of methyl groups on the chromanol ring (Figure 1).

Though tocotrienols were characterized more than five decades ago, the majority of their biological attributes were only revealed over the last 15 years. Now, tocotrienol research has gained significant momentum and attention [39]. The latest developments in the area of vitamin E research clearly demonstrate that the members of the vitamin E family are not redundant in relation to their biological properties and functions [33]. It has been found that α-, γ- and δ-tocotrienols have functions involved with disease treatment and health maintenance clearly distinct from those of tocopherols [34]. The various isoforms of tocotrienols differ in their biological properties; some earlier studies have demonstrated that there may be a 30-fold difference in the capacity of δ-, γ- and α-tocotrienol to inhibit the biosynthesis of cholesterol [34]. The antioxidant, anti-inflammatory and cholesterol-lowering attributes of tocotrienols may prevent diabetes, cancer, neurodegenerative and cardiovascular disorders [59]. Additional studies have suggested that α-tocotrienol is most neuroprotective [29]; δ-tocotrienol can target prostate cancer [60]; δ-tocotrienol treatment is effective against pancreatic carcinoma [61].

Tocols and their several promising derivatives have been extensively investigated and recently reviewed for their radioprotective efficacy [13,15,28,62,63,64]. Tocopherol succinate, δ-tocotrienol and GT3 have comparable radioprotective efficacy and appear better than other tocols as radioprotectants [40,65,66,67]. GT3 is an inhibitor of 3-hydroxy-3-methylglutaryl-coenzyme A (HMG-CoA) reductase [68] and demonstrated radioprotective efficacy in NHPs in a recently published study [69]. GT3 is being developed as a radioprotector for military and civilian use at AFRRI in collaboration with the University of Arkansas. Here, we discuss the work being conducted to develop GT3 as a radiation countermeasure.

## 5. Radioprotective Efficacy of γ-Tocotrienol (GT3) in the Mouse Model

**Figure 2:**
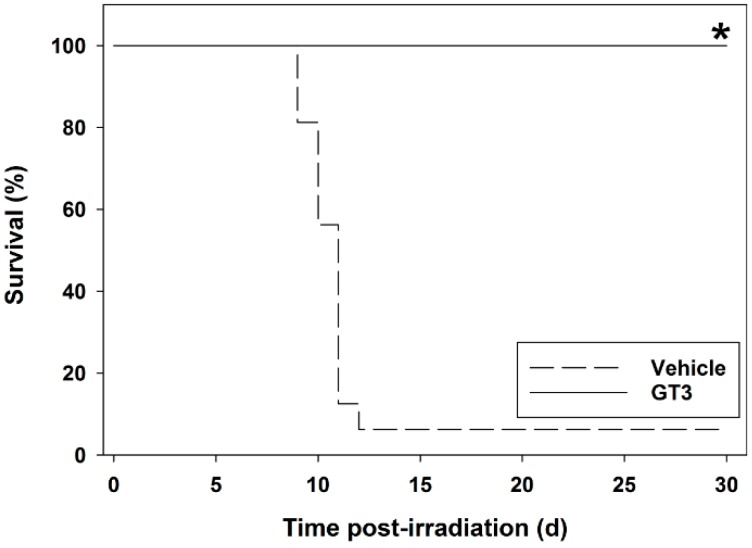
Radioprotective efficacy of subcutaneously-administered γ-tocotrienol (GT3) in irradiated CD2F1 strain male mice. Two groups of mice were administered either GT3 (200 mg/kg) or vehicle subcutaneously 24 h prior to whole body irradiation with 9.2 Gray (Gy) (dose rate 0.6 Gy/min). Mice were observed for 30 days. Mice treated with GT3 had a significantly higher survivor rate compared to the vehicle control group (*n* = 16 each group). * Denotes significant difference between the GT3 and vehicle control groups (*p* < 0.05) [70].

GT3’s antioxidant activity was a compelling reason to evaluate it for its radioprotective efficacy. Studies have shown that a single subcutaneous administration prior to whole body irradiation is capable of dramatically decreasing radiation injury in several organ systems, including the GI, the hematopoietic system and the vascular system. In fact, after a radiation dose that is uniformly lethal within 14 days, mice receiving a single dose of GT3 24 h prior to irradiation show near 100% long-term survival (Figure 2). Comparatively, GT3 appears to be the most promising tocol tested as a radiation countermeasure to date.

### 5.1. Radioprotection against Hematopoietic Injury

**Figure 3:**
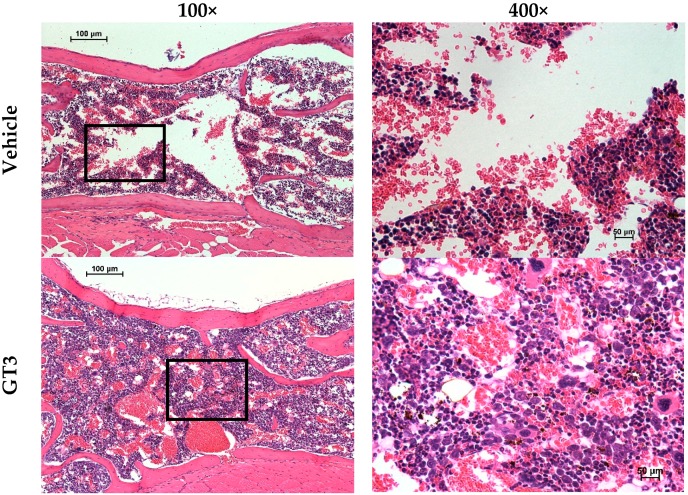
The efficacy of GT3 administration on sternal cellularity and megakaryocytes in mice exposed to ionizing radiation. Two groups of mice were administered subcutaneously the vehicle or GT3 (200 mg/kg) 24 h prior to irradiation. Mice were exposed to 9.2 Gy (0.6 Gy/min) whole body γ-radiation. Sterna from mice were harvested 6 h after irradiation. Cross-sections of sterna were processed for tissue fixation, and sternal bone marrow cells were stained (H&E) to score cellularity. Representative areas of cross-sections are shown (**left** panels, 100×; **right** panels are the marked enlarged area from respective left panels of each photomicrograph at 400× magnification) [70].

GT3 has been demonstrated to significantly enhance mouse survival through ameliorating the radiation-induced injuries of the hematopoietic and GI systems [40,71,72]. In the CD2F1 strain mouse model, the GT3 dose reduction factor (DRF) has been estimated to be 1.29. Additional studies have suggested 24 h prior to irradiation to be the most effective time for administration. This may be due to the induction of important hematopoietic cytokines. The optimal dose of GT3, 200 mg/kg, accelerated hematopoietic recovery (Figure 3) and improved peripheral blood profiles (total white blood cells, platelets, reticulocytes, neutrophils and monocytes,) [40]. Colony-forming assays on sorted hematopoietic stem cells suggested that whole body irradiation reduced the total number of colonies in irradiated mice compared to the unirradiated group (naive mice). Irradiated mice treated with GT3 had higher numbers of progenitor colonies, suggesting preservation of the self-renewable capacity of hematopoietic stem cells [73]. Histopathological evaluation of mouse sternum shows that GT3-treated animals have more myeloid regenerative microfoci, as well as megakaryocytes and had better cellularity compared to vehicle-treated and irradiated control animals at 7 and 13 day after whole body irradiation. GT3 treatment resulted in significantly reduced numbers of micronucleated erythrocytes, suggesting that GT3 protects hematopoietic tissue by preventing persistent DNA damage in the hematopoietic stem and progenitor cells [73].

### 5.2. Radioprotection against Gastrointestinal and Vascular Injuries

GT3 has demonstrated the ability to ameliorate GI radiation injury by improving the survival of intestinal crypt cells, the recovery of the intestinal mucosal surface area, the acceleration of soluble endothelial function markers, as well as the reduction of the vascular oxidative stress in a manner independent of HMG-CoA reductase after irradiation [72]. GT3’s ability to decrease radiation-induced oxidative stress was reversed by mevalonate. These findings may have significant implications for the future development of GT3, particularly pertaining to organ injury where vascular damage is presumed to play an important mechanistic role (*i.e*., lung and intestine). Tocotrienols accumulate in the small intestine, as well as colon to greater levels than tocopherols, which may also aid in their ability to reduce GI injury [74]; in fact, GT3 concentrates in endothelial cells at concentrations 30- to 50-times greater than α-tocopherol [75].

GT3 reduces post-irradiation vascular peroxynitrite production through inhibition of HMG-CoA reductase. The inhibitors of this kind mediate their efficacy by endothelial nitric oxide synthase, with tetrahydrobiopterin (BH4) as an important cofactor. The effects of irradiation on the bioavailability of BH4 have been investigated in mice, as well as those of GT3 on BH4 metabolism [71].

Results of a recent study show that concentrations of BH4 in lung decreased compared to baseline values 3.5 days post-irradiation; however, the treatment with GT3 reversed this effect. Both GT3, as well as BH4 supplementation significantly inhibited the production of vascular peroxynitrite at 3.5 days post-irradiation and increased bone marrow colony formation. GT3 administration modulated apoptosis of endothelial cells, reduced guanosine triphosphate cyclohydrolase-1 (GTPCH) feedback regulatory protein, known as GFRP, and resulted in reduced GFRP-GTPCH binding. These results suggest reduction in the bioavailability of BH4 in the early post-irradiation period. They also suggest that exogenous administration of BH4 reduces post-irradiation vascular oxidative stress. GT3 may produce some of its valuable effects on free radical production after irradiation partly by offsetting the reduction in BH4, potentially by reducing the expression of GFRP.

GT3 has also been shown to upregulate A20, an anti-inflammatory enzyme and inhibitor of nuclear factor-κB (NF-κB), which leads to basal activation of NF-κB. GT3 treatment increased phosphorylation of translation initiation factor-2, inhibitor of κBα (IκBα) and Jun amino-terminal kinase. The basal activation of NF-κB may lead to the upregulation of protective enzymes and other proteins, which may result in radioprotection [76,77].

## 6. Cytokine Induction by GT3

The effects of GT3 on the hematopoietic system have been evaluated by measuring several cytokines, chemokines and growth factors by multiplex Luminex. Treatment of mice with GT3 resulted in high levels of granulocyte colony-stimulating factor (G-CSF), interleukin-1 α (IL-1α), IL-1β, IL-6, IL-12p70, IL-17, macrophage inflammatory protein-1α and keratinocyte chemoattractant levels. The levels of G-CSF significantly increased within 12 to 24 h after GT3 injection [70,78]. Most of these cytokines were upregulated in the presence, as well as in the absence of irradiation. Time-course analysis of G-CSF and IL-6 was interesting; both cytokines were induced transiently after injection with GT3 and returned to pre-injection levels by 48 h. Where the peak G-CSF concentration was observed between 12 and 24 h after GT3 injection, the highest levels of IL-6 were observed between 6 and 12 h after GT3 injection. These findings suggest that GT3 induces high levels of G-CSF, inflammatory cytokines and chemokines within 24 h of administration. These findings may help explain why survival studies indicate that 24 h prior to irradiation was the ideal administration time to provide the optimal survival benefit; GT3 may induce important hematopoietic cytokines and cytokines during that time window. These results also demonstrate the role of GT3-mediated G-CSF induction in protecting animals from radiation-induced hematopoietic injury, particularly neutropenia.

### 6.1. Efficacy of GT3 Is Mediated through Granulocyte Colony-Stimulating Factor (G-CSF) Production in the Mouse Model

G-CSF and other cytokines have demonstrated the ability to prevent radiation-induced neutropenia and H-ARS in several animal models [13,79,80]. G-CSF induced in response to irradiation or administration of some radiation countermeasures plays an important role in radioprotection [9,81]. In a recent study, we investigated the role of G-CSF induction in mice for the protection afforded by GT3. Mice were exposed to 9.2 Gy cobalt-60 γ-radiation and evaluated by neutralizing G-CSF with an antibody; the mice were then observed for survival for 30 days. The neutralization of G-CSF by the antibody was confirmed in unirradiated, as well as irradiated mice. The results demonstrated that G-CSF antibody administration completely inhibited GT3-induced G-CSF in peripheral blood of irradiated and unirradiated mice, but did not decrease levels of any other cytokine stimulated by GT3. G-CSF neutralization by this method completely abrogated the radioprotection provided by GT3 [70]. Similar to GT3, we have demonstrated abrogation of radioprotective efficacy of several other radiation countermeasures inducing high levels of G-CSF by its antibody administration in a murine model [41,82,83,84].

### 6.2. Mobilization of Mouse Progenitors by GT3-Induced G-CSF

Tocols have a bone marrow progenitor-mobilizing ability, and as discussed above, GT3 has been demonstrated to induce G-CSF, which is known to mobilize progenitors into the peripheral circulation [70,78,85]. In fact, GT3 has been shown to induce higher levels of all three types of progenitor cells, hematopoietic (lineage negative (Lin^−^), stem cell factor positive (c-Kit^+^)), stromal (Lin^−^, cluster of differentiation 29 positive (CD29^+^), CD105^+^) and endothelial (Lin^−^, CD34^+^, endothelial progenitor cell marker positive (Flk^+^)), compared to vehicle, into peripheral circulation; AMD3100 (Plerixafor, Mozobil) enhances GT3’s capabilities of induced mobilization [86].

In order to investigate the radiomitigative potential of progenitors mobilized by GT3, experiments were performed where mice exposed to a high radiation dose (11 Gy) received administration of GT3-mobilized progenitors from donor mice. Administration of blood or mononuclear cells from peripheral circulation significantly enhanced survival of the lethally-irradiated mice [87]. Furthermore, the irradiated mice receiving peripheral blood mononuclear cell administration from GT3-injected donor mice retained GI structural integrity and had significantly reduced incidence of bacterial translocation from the GI tract to peripheral blood circulation and other organs. This suggests that such blood transfusions or mononuclear cell administration also provide GI recovery in addition to hematopoietic recovery in irradiated mice. These benefits were significantly abrogated by the administration of G-CSF antibody to the donor mice. Such antibody administration neutralized the GT3-induced G-CSF, ultimately inhibiting the mobilization of sufficient bone marrow progenitor cells for optimal mitigation of radiation injury. In brief, the capacity of GT3 to mobilize bone marrow progenitors can be used for the treatment of radiation injuries. Mobilization of bone marrow progenitors into peripheral circulation by GT3 suggests that GT3 can be substituted for G-CSF and vascular endothelial growth factor to mobilize progenitors in the peripheral circulation. Such a treatment option appears to be really attractive based on preliminary studies in the mouse model with another tocol, tocopherol succinate [85,88,89].

## 7. Combination of GT3 with Other Agents for Enhancing Its Radioprotective Efficacy

During the recent past, a few agents with different modes of action have been tested in combination with GT3 to further enhance its radioprotective efficacy in the mouse model; it appears to be an encouraging strategy.

### 7.1. Radioprotective Efficacy of GT3 and Pentoxifylline (PTX) Combination

The radioprotective efficacy of GT3 also stems from its capacities to concentrate in endothelial cells and inhibit the HMG-CoA reductase activity, similar to the drug class statins. HMG-CoA reductase inhibitors are often used in the treatment of hyperlipidemia. Such inhibitors have significant vasculoprotective, anti-inflammatory, as well as anti-fibrotic effects. These activities are mediated by endothelial nitric oxide synthase [90]. Even though it works through a different pathway than HMG-CoA reductase inhibitors, PTX has similar anti-fibrotic, antioxidant, anti-inflammatory and vasculoprotective effects. The combined effects of GT3 and PTX have been studied on whole body irradiation-induced acute hematopoietic, GI and vascular injuries and subsequent mortality. Nitric oxide synthase-deficient mice were used to investigate whether protection against lethality provided by GT3, PTX or the combination of the two agents required the presence of endothelial nitric oxide synthase. Evidence indicates that GT3 in combination with PTX is more effective in improving survival after whole body radiation exposure compared to pre-treatment with GT3 alone [91]. The data also suggest that GT3 plus PTX administration may modulate hematopoietic recovery by the induction of hematopoietic factors. Combination therapy did not reduce post-irradiation GI injury or vascular peroxynitrite production compared to treatment with GT3, and the protective effect does not appear to depend on nitric oxide synthase.

Additional studies were conducted to systematically determine the most efficacious schedule and dose of PTX administration, as well as the DRF of the combination. To determine the mechanism of synergistic radioprotection provided by GT3-PTX, mevalonate or calmodulin was co-administered to reverse the effect of GT3 on HMG-CoA reductase or PTX on the inhibition of phosphodiesterase, respectively. Calmodulin administration results indicate that the increase in radioprotective efficacy provided by the GT3-PTX combination was due to phosphodiesterase inhibition [92]. Calmodulin administration also reversed PTX’s ability to increase cyclic adenosine monophosphate and calcium signaling, which also play a role in PTX’s ability to increase the radioprotective efficacy of GT3.

### 7.2. Radioprotective Efficacy of GT3 and Amifostine Combination

Recently, we have shown using the mouse model that radioprotective doses for amifostine occur between 25 and 50 mg/kg. The mature and lineage-restricted progenitor cells were more responsive to the protective effects of amifostine low dose compared to the multipotential (more primitive) progenitors [93]. Low-dose GT3 has been evaluated in conjunction with low doses of amifostine prophylaxis against whole body irradiation with the hopes of minimizing the adverse drug-related side-effects of amifostine administration. An additive radioprotective action was predicted, as each agent possesses a different mode of action and distinct toxicity profiles. Male CD2F1 mice received amifostine and/or GT3 before exposure to 9.2 Gy cobalt-60 γ-irradiation. Survival studies showed that the combination treatments resulted in significantly higher survival compared to either single treatment, even when single treatments provided insignificant survival benefits compared to the vehicle control [94]. Serum cytokines were analyzed by multiplex Luminex, which confirmed the prior work indicating that GT3 significantly induces G-CSF. Amifostine did not induce G-CSF in either the *in vivo* or *in vitro* studies. The approach utilizing prophylactic combination of amifostine and GT3 shows great promise and should be investigated further as a potential countermeasure for ARS.

### 7.3. Contribution of Thrombomodulin to GT3-Mediated Lethality Protection

A robust upregulation of thrombomodulin is a prominent feature of all HMG-CoA reductase inhibitors, including GT3. The efficacy of GT3 as a radiation countermeasure has been shown to be dependent on its ability to increase endothelial cell thrombomodulin *in vitro* [95]. In this study, the levels of G-CSF appeared to be unrelated to hematopoietic recovery, suggesting that G-CSF does not mediate the thrombomodulin-dependent effect of GT3. These data indicate that synergistic or additive radioprotection can be achieved by using GT3 and other HMG-CoA reductase inhibitors in combination with thrombomodulin and/or activated protein C.

## 8. Radioprotective Efficacy of GT3 in Nonhuman Primates (NHPs)

GT3 is capable of providing protection to mice against radiation doses close to those doses capable of inducing the full-fledged GI syndrome, meriting its further development in the NHP model. Three drug doses (9.375, 18.75 and 37.5 mg/kg) administered subcutaneously were used for pharmacokinetic studies based on efficacy and toxicity studies conducted in the mouse model. For each dose, the *C*_max_ (maximum blood plasma concentration for GT3), the *T*_max_ (time at which maximum blood plasma concentration was reached), *T*_1/2_ (half-life of GT3), AUC (area under the curve), clearance, and MRT (mean retention time) were determined. It was found that, as the dose increased, so did the area under the curve and half-life [69].

**Figure 4:**
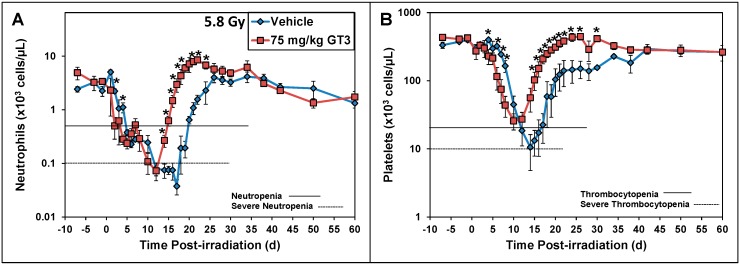
Effects of radiation exposure and GT3 treatment on the levels of neutrophils and platelets in peripheral blood of irradiated nonhuman primates (NHPs). Animal received GT3 (75 mg/kg, red) 24 h prior to irradiation. The vehicle control is colored blue. NHPs were irradiated with 5.8 Gy (0.6 Gy/min, cobalt-60 γ-radiation), and blood samples were collected at various time points in relation to irradiation. Neutrophils (**A**) and platelets (**B**) were analyzed with the Advia 120 cell analyzer. The mean and the standard error of the mean are displayed. The significant differences between GT3- and vehicle-treated groups are marked with * (*p* < 0.05) [69].

The radioprotective efficacy of GT3 was tested in the NHP model, with three different doses of cobalt-60 whole body irradiation (5.8 Gy, 6.5 Gy and 7.2 Gy; dose rate 0.6 Gy/min). Two different doses of GT3 (37.5 and 75 mg/kg) were tested against 5.8 Gy. For the remaining two doses of radiation, only one dose of GT3 (37.5 mg/kg) was evaluated. The efficacy study results demonstrate promising hematopoietic recovery after administration of GT3 to irradiated NHPs, by significantly decreased neutropenia and thrombocytopenia compared to vehicle-treated irradiated controls (Figure 4) [69]. In GT3-treated, irradiated animals, neutrophil and platelet levels decrease early, but recover quickly. Consistent with the improvement in neutropenia, thrombocytopenia and cytopenia, lymphocyte counts in GT3-treated animals (5.8 Gy, 75 mg/kg; 6.5 and 7.2 Gy, 37.5 mg/kg) were significantly higher than in controls at a few time points [69]. It is important to note that low lymphocyte counts have been reported to be the best predictor of radiation lethality for humans in the Medical Treatment Protocols for Radiation Accident Victims [96]. In our study, the survival benefit from GT3 treatment of irradiated animals 60 days post-irradiation did not reach statistical significance; this may be due to the small sample size of four or eight animals per group. We are conducting a study using a larger sample size, and this study is supported by the Congressionally Directed Medical Research Programs of the U.S. Department of Defense.

Though the GT3 study used a similar NHP model as reported earlier for other radiation countermeasures [97,98,99,100,101,102,103,104], this study excluded supportive care (antibiotics, blood products and intravenous fluids) to allow for a better demonstration of the radioprotective efficacy of GT3 in a situation that closely simulates the conditions that would be expected after a mass casualty scenario when these additional products and appropriate infrastructure facilities would be limited. The efficacy offered by a single dose of GT3 on hematopoietic recovery without any supportive care in NHP was comparable to, and at times better than, 18 doses (one dose a day) of Neupogen (or two doses of Neulasta) with full supportive care [97,104].

Our study data indicate that GT3 administration resulted in better hematopoietic recovery with respect to neutropenia, thrombocytopenia and cytopenia after 5.8 and 6.5 Gy radiation than recombinant human IL-12 (HemaMax) without supportive care after 6.7 or 7.0 Gy (Theratron 1000 cobalt-60 source) irradiation [100,101,105]. Comparable results with respect to improving neutropenia and thrombocytopenia have also been demonstrated using another growth factor, granulocyte-macrophage colony-stimulating factor, in partial body radiation-exposed NHPs (8 Gy, tibiae shielded with lead walls) [99].

CBLB502 (Entolimod), a toll-like receptor 5 ligand (*Salmonella enterica* serovar *Dublin* truncated flagellin) has been extensively studied in NHPs. A single dose of Entolimod administered either before or after lethal whole body irradiation significantly improved animal survival, neutropenia, reduced radiation damage to the GI and hematopoietic systems and improved tissue regeneration [102,106]. The radiomitigator 5-androstenediol, a naturally-occurring adrenal steroid hormone, significantly reduced the duration of thrombocytopenia and neutropenia in NHP exposed to 4 Gy or 6 Gy cobalt-60 γ-radiation; both radiation doses are capable of inducing H-ARS [103,107]. Ultimately, the hematopoietic recovery afforded by GT3 in NHPs was comparable to each, G-CSF, PEGylated G-CSF, granulocyte-macrophage colony-stimulating factor, CBLB502 and 5-androstenediol.

Citrulline is a well-known end product of glutamine metabolism and has been identified as a biomarker for radiation injury in small intestine. This marker is an indicator of GI epithelial cell loss [108,109]. The circulating citrulline concentration is inversely related to enterocyte mass [110]. Recently, it has been demonstrated that whole body irradiation in the hematopoietic dose range is capable of inducing substantial GI injury in NHPs [111]. The GT3-treated NHPs (37.5 mg/kg) irradiated with 7.2 Gy had higher citrulline concentrations on a few time points post-irradiation compared to the vehicle-administered group, suggesting quicker recovery of small intestine in GT3-treated animals [53].

Lethal doses of whole body irradiation exposure induce polymicrobial sepsis as a result of intestinal epithelium injury and subsequent bacterial translocation. We studied gut bacterial translocation to peripheral circulation in GT3-treated and untreated irradiated NHPs. Although we found several bacterial species in peripheral circulation of irradiated NHPs, there was no significant difference between vehicle control and GT3-treated groups exposed to different doses of radiation [69].

## 9. Mechanisms of Action

**Figure 5:**
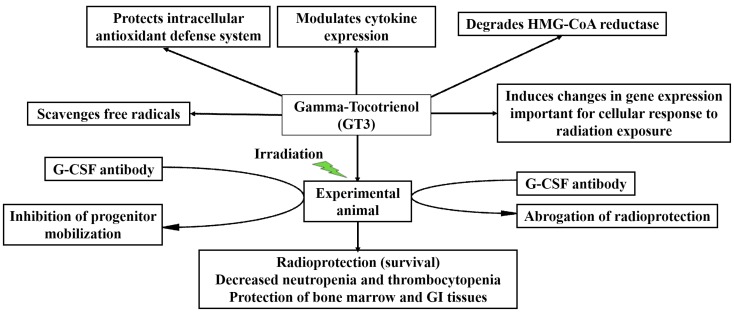
Potential mechanism of action for GT3 for its radioprotective efficacy.

The exact mechanisms for the radioprotective efficacy of GT3 are not well understood (Figure 5). A recent *in vitro* study investigated the gene expression profile in human epithelial cells and compared GT3 to two other tocopherols of the vitamin E family, γ-tocopherol and α-tocopherol. After 24 h of treatment with GT3, γ-tocopherol or α-tocopherol, endothelial cells were harvested, and RNA was extracted for conducting genome-wide sequencing for gene expression using Illumina. GT3 was found to be more effective than both γ-tocopherol, as well as α-tocopherol in modulating changes in the gene expression profile [112]. Several pathway genes were affected, including those responsible for hematopoiesis, angiogenesis, DNA damage stimuli, cell cycle, cell proliferation, cell death, oxidative stress and response to DNA damage. The results from this study need to be further investigated to understand the differentially-affected gene ontology. Such a study will shed light on the protection of endothelial cells by GT3.

### 9.1. Antioxidant Properties of GT3

There are conflicting results in regards to the antioxidant activity of tocotrienols *versus* tocopherols. Some recent studies have suggested that tocotrienols have better antioxidant activity than tocopherols [35,36,37,38]. One study suggests that the antioxidant potency of tocotrienols is 1600 times superior to α-tocopherol [113]. These reports suggest that the difference in antioxidant efficacy may lie in tocotrienol’s ability to penetrate into the tissue more quickly due to its unsaturated aliphatic tail. However, other studies were unable to confirm such a difference in antioxidant potential between tocotrienols and tocotrienols [114]. These apparently conflicting data may be a result from the circumstances under which the assays are performed. The radioprotective efficacy of tocopherol succinate, δ-tocotrienol and GT3 are comparable (DRFs of all three agents between 1.25 and 1.30), but these agents appear to be better than α-tocopherol. Such observations suggest that superior antioxidant activity may not reflect improved radioprotective efficacy, and other unknown factors may also be important.

In a recent study, it was observed that GT3 is taken up by renal proximal tubular cells, reduces mitochondrial dysfunction, restores adenosine triphosphate levels, blocks free radical bursts and prevents renal proximal tubular cell lysis and death after oxidant-induced injury. Mitochondria are a major target of protective actions of GT3 in injured renal proximal tubular cell [115]. GT3 improves mitochondrial respiration, coupling and mitochondrial membrane potential (ΔΨ_m_) and maintains oxidative phosphorylation and adenosine triphosphate levels in injured renal proximal tubular cells.

### 9.2. Effects of GT3 on 3-Hydroxy-3-methylglutaryl-coenzyme A (HMG-CoA) Reductase

HMG-CoA reductase is an important enzyme in cholesterol biosynthesis and catalyzes the rate-limiting step during cholesterol biosynthesis. The addition of tocopherols in the diet of laboratory animals resulted in increased levels of total, as well as low-density lipoprotein cholesterol levels in peripheral circulation, whereas the addition of tocotrienols lowered these levels [116]. GT3 and other HMG-CoA reductase inhibitors, like statin, inhibit HMG-CoA reductase by different mechanisms. GT3 induces degradation of HMG-CoA reductase in the ubiquitin-proteasome pathway rather than, as statins, inhibiting the enzyme itself [117,118]. These findings suggest a probable mechanism for the observed cholesterol lowering efficacy of tocotrienols in animals and humans. When tocotrienols were orally administered to mice, they appear faster, but at lower levels in the blood plasma compared to tocopherols [119]. The faster appearance of tocotrienols in the circulation, as well as their low level sustained release imply that GT3 may be a better therapeutic agent than tocopherols.

### 9.3. Anti-Apoptotic Activity of GT3

Since apoptosis is a major mediator of radiation-induced injury, it is important to understand how GT3 modulates apoptosis regulatory pathways in intestinal cells after exposure to radiation doses capable of inducing GI syndrome. A recent study analyzed the intestinal epithelium at 4 and 24 h after 11 Gy γ-irradiation by evaluating the expression of 84 apoptosis-related genes. Prophylactic GT3 administration inhibited the pro-apoptotic protein, Bak1, and enhanced the anti-apoptotic proteins, Bag3, Rnf7 and Tsc22d3/Gilz [120].

## 10. Conclusions and Future Direction

In the era of the heightened risk of radiological/nuclear terrorism or power plant accidents, appropriate medical contingency plans are critical to save human lives. The availability of effective radiation countermeasures is the key to preparedness success. GT3 appears to be a promising radiation countermeasure for ARS, as it has shown particularly potent radioprotective efficacy in relevant animal models [15,28,69]. GT3 has been shown to protect endothelial cells from radiation damage due to its antioxidant properties, inhibition of HMG-CoA reductase and improvement of BH4 bioavailability [71]. GT3 induces several changes known to be of vital importance in the cellular and molecular responses to radiation exposure, such as radiation-induced oxidative stress, damage to DNA, regulation of apoptosis, cell proliferation, hematopoiesis and angiogenesis [112]. GT3-mediated protection of intestinal cells occurs by downregulation of pro-apoptotic factors, as well by as upregulation of anti-apoptotic factors [120]. Furthermore, GT3 treatment combined with PTX increased post-irradiation survival over that of GT3 alone by a mechanism that may depend on the induction of hematopoietic stimuli [91].

Though much is known about the potential mechanism by which GT3 works as a radioprotector, there are ongoing investigations to further determine the mechanisms by which single injections of GT3 elicit such a powerful radioprotective response. A clinical trial is underway to investigate GT3’s efficacy in combination with PTX for delayed radiation enteropathy. In addition to its use as a medical countermeasure for ARS, GT3 may find additional use in reducing radiation injury in radiotherapy patients. Even though little is known about the effects of GT3 on tumor cells during radiotherapy, it is suggested that GT3 may sensitize tumor cells to radiation exposure and chemotherapeutic agents [121,122,123]. Additional studies are required to confirm that GT3 does not in fact protect tumor cells when exposed to radiation. As far as the safety of GT3 in humans is concerned, there are studies from other areas that have demonstrated that GT3 supplementation is well tolerated in humans [124,125].

GT3 administered orally was minimally effective as a radioprotective agent in contrast to the subcutaneous administration of GT3 in mice. Such results suggest that the brief residence of GT3 administered orally is not enough to provide radioprotection in mice. Tocotrienols bind with α-tocopherol transfer protein for transport from the liver to the peripheral circulation. The shorter residence of tocotrienols may be due to their limited binding ability to the transfer protein for transport. For a radioprotective agent to be optimally effective through the oral route, the agent should have a large area under the curve and a longer half-life. Thus, it is desirable to develop an analog of GT3 with improved pharmacokinetic properties. Effort is being made to develop a compound, tocoflexol, with a more flexible tail to improve the binding to α-tocopherol transfer protein, thereby maintaining the superior bioactivity of the tocotrienols while achieving a half-life and area under the curve comparable to that of the tocopherols [126].

One would expect that hospital-based medical care facilities to be limited under a mass casualty scenario. Therefore, an optimal radiation countermeasure should be efficacious in the absence of intensive hospital-based medical care. The results of GT3’s radioprotective efficacy in NHPs without supportive care is significant, as the radiation doses used in our study are equivalent to the lethal human dose. GT3 is regarded by the U.S. FDA as GRAS when used as a food additive (*i.e*., used orally) at lower doses than contemplated for use as a radiation countermeasure. This agent needs to be developed following the U.S. FDA Animal Efficacy Rule; phase I safety studies are required to evaluate the safety and pharmacokinetic profiles of GT3. The human toxicity and comprehensive animal studies will allow finding the predictive dose of GT3 efficacy in humans. Since GT3 is a prophylactic agent (radioprotector), it is more suitable for military personnel, first responders and individuals anticipating radiation exposure. Currently, GT3 efficacy and the mechanism of action are being investigated using the NHP model with support from Congressionally Directed Medical Research Programs, U.S. Department of Defense, for further development.

## Abbreviations

ΔΨ_m_	Mitochondrial membrane potential
ARS	Acute radiation syndrome
BH4	Tetrahydrobiopterin
CD	Cluster of differentiation molecule
c-Kit^+^	Stem cell factor positive
DRF	Dose reduction factor
Flk^+^	Endothelial progenitor cell marker positive
G-CSF	Granulocyte colony-stimulating factor
GFRP	GTPCH feedback regulatory protein
GI-ARS	Gastrointestinal acute radiation syndrome
GRAS	Generally recognized as safe
Gy	Gray
GTPCH	Guanosine triphosphate cyclohydrolase 1
H-ARS	Hematopoietic acute radiation syndrome
HMG-CoA	3-Hydroxy-3-methylglutaryl-coenzyme A
IL	Interleukin
Lin^−^	Lineage negative
IκBα	Inhibitor of κBα
NF-κB	Nuclear factor-κB
PTX	Pentoxifylline
U.S. FDA	United States Food and Drug Administration
